# Interaction mechanism of oseltamivir phosphate with bovine serum albumin: multispectroscopic and molecular docking study

**DOI:** 10.1186/s13065-024-01232-0

**Published:** 2024-07-05

**Authors:** Jing Yu, Jian-Ming Liu, Hui-Yi Chen, Wei-Ming Xiong

**Affiliations:** 1 School of Chemistry and Civil Engineering, Shaoguan University, Shaoguan, 512005 China; 2 School of Physical Science & Technology, Guangxi University, Nanning, 530004 China

**Keywords:** Oseltamivir phosphate, Bovine serum albumin, Multispectroscopic, Molecular docking

## Abstract

Oseltamivir phosphate (OP) is an antiviral drug with potential risks to human health due to overuse, leading to serious consequences such as gastrointestinal disturbances, abnormal neuropsychiatric symptoms, and sudden death. Therefore, gaining an in-depth understanding of its interaction with proteins is crucial. We investigated the interaction between OP and bovine serum albumin (BSA) utilizing multispectral methods (i.e., fluorescence, ultraviolet absorption, circular dichroism) combined with molecular docking techniques. Fluorescence spectroscopy indicated that OP quenched BSA fluorescence by forming the OP-BSA complex. The Stern-Volmer constants (*K*_SV_) between OP and BSA were determined to be 3.06 × 10^3^ L/mol, 2.36 × 10^3^ L/mol, and 1.86 × 10^3^ L/mol at 293 K, 298 K, and 303 K, respectively. OP occupies exclusively one binding site on BSA, and the fluorescent probe displacement measurements revealed that this is BSA site I. Thermodynamic data (*∆H*, ∆*S*, and ∆*G*) obtained by fitting the van’t Hoff equation were − 77.49 kJ/mol, -176.54 J/(mol∙K), and − 24.88 kJ/mol, respectively, suggesting that hydrogen bonding and van der Waals forces mainly participate in OP-BSA complex stabilization. Moreover, the reaction occurs spontaneously at room temperature. Synchronous fluorescence spectra indicated that OP interacts with tryptophan residue of BSA. The results of ultraviolet (UV) and 3D fluorescence spectroscopy indicated that the OP-BSA complex formation altered the microenvironment around amino acid residues. Circular dichroism spectra revealed that the addition of OP decreased the α-helix content of BSA by 7.13%. Docking analysis confirmed that OP binds to BSA site I through hydrogen bonding with amino acids VAL342, SER453, and ASP450. Finally, ADMET studies were conducted to explore the pharmacokinetics of OP as an antiviral drug.

**Supplementary Information:**

The online version contains supplementary material available at 10.1186/s13065-024-01232-0.

## Introduction

**Fig. 1:**
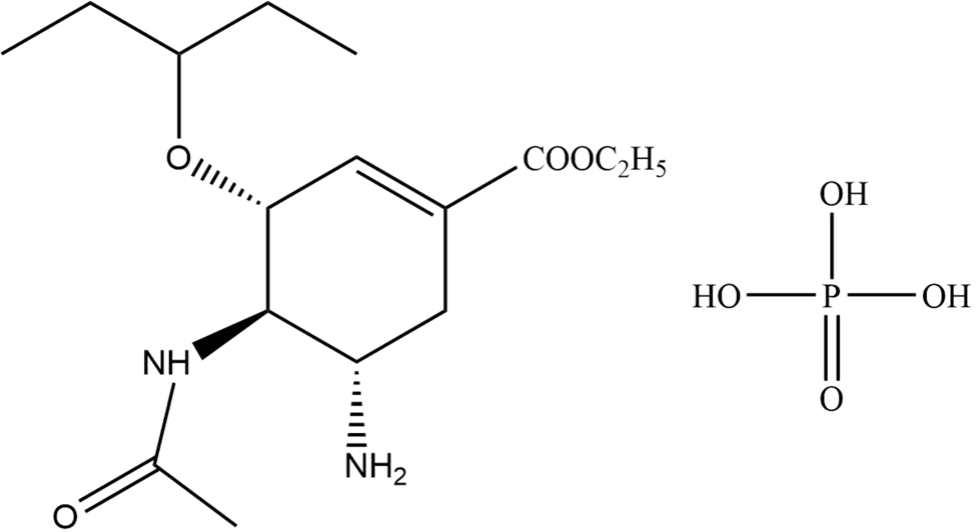
Schematic diagram of OP molecular structure

The antiviral drug Oseltamivir phosphate (C_16_H_31_N_2_O_8_P, (3R,4R,5 S)-4-acetamido-5-amino-3-(1-ethyl epoxy)-1-cyclohexene-1-carboxylic acid ethyl ester phosphate, OP, see Fig. 1) is a neuraminidase inhibitor that acts by blocking the release and transmission of viral progeny from infected cells [1, 2]. In August 2016, the drug was FDA-approved for the treatment and prevention of influenza A (including pandemic H1N1) and B virus infections [3]. It also inhibits tumor angiogenesis, growth and metastasis [4]. Its applications to ester-loaded drug delivery systems, such as ionic complexes for palatability modulation, swellable hydrogel systems for modified release, and liposome-encapsulated oseltamivir salts in dry powder formulations for pulmonary delivery have been reported in the literatures [5, 6]. In addition, it can also be used to limit the spread of infection in high-risk populations, as recommended by the World Health Organization (WHO) [7]. However, researchers have demonstrated through numerous clinical trials that OP use did not reduce the risk of hospitalization but increased the incidence of gastrointestinal adverse events [8, 9]. Studies have shown that doses of OP may cause symptoms such as nausea and vomiting in humans [10], and overdose of OP may lead to serious consequences such as gastrointestinal disturbances [11], abnormal neuropsychiatric symptoms [12], and sudden death [13, 14]. Therefore, it is necessary to evaluate its toxicity and safety.

Drugs deliver their desired therapeutic effects by interacting with proteins in the targeted diseased tissue or site of action [15]. The binding of drugs to proteins in vivo significantly influences their therapeutic properties [16]. If a drug has a strong binding capacity, this may prolong its residence time in the body, thereby increasing the likelihood of adverse side effects. On the contrary, a drug with weak binding capacity may face issues related to shorter half-life and early excretion from the body, making it less effective [17]. The therapeutic effectiveness of a drug molecule is impacted by its ability to bind to proteins, which can also influence its toxicity and stability during therapy [18]. This suggests that reversible binding of drugs to proteins determines pharmacokinetic parameters such as metabolism, distribution, transport, and excretion of drugs [19–22]. Therefore, exploring the binding mechanisms of drugs to proteins is crucial.

Among many proteins, bovine serum albumin (BSA) has a three-dimensional structure similar to that of human serum albumin (HSA), so it is often used as an ideal model system for in vitro studies of interactions with drugs [23, 24]. BSA is a versatile protein with multiple binding sites and several carboxylic and amino groups that can interact with small molecules [25–27]. This makes it an effective transport protein that can create stable complexes with various external and internal ligands, which may influence the effectiveness, distribution and storage of small molecule drugs [28–30]. Due to the potential risks of OP, it is of vital importance to conduct explorations related to OP’s association with SA so as to elucidate the relationship between OP and human health.

In this study, we conducted a comprehensive analysis of the binding mode and interaction strength between OP and BSA by employing various spectroscopic methods along with molecular docking. The experimental techniques provided insights into the binding capacity, quenching type, force of action, binding site, secondary structure changes, and OP-BSA complex properties. Meanwhile, molecular simulations rationalized the binding mode by highlighting the amino acid residues that interact with OP and estimated the binding free energy of the OP-BSA complex. Additionally, OP was evaluated by exploring its ADMET (absorption, distribution, metabolism, excretion and toxicity) properties in silico. Through these studies, we aim to understand the effect of OP on BSA and provide valuable risk aversion data for the clinical application of this antiviral drug. Building on this research, drug-protein interactions can be optimized to achieve the development of more effective and targeted antiviral drugs.

## Experimental

### Materials

BSA (M = 66.430 kDa, purity 96-99%, CAS# 9048-46-8) was acquired from Shanghai Aladdin Biochemical Technology Co., Ltd. Phosphate buffer solution (PBS, 0.02 mol∙L^− 1^) with a pH of 7.43 was prepared using NaH_2_PO_4_ and Na_2_HPO_4_ (p.a.). The BSA solution in PBS (1 µM) was then made and stored at 4 ℃. Prior to the experiment, the OP (CAS# 204255-11-8) stock solution in anhydrous ethanol (1 mM) was made. Ultrapure water was used in all experiments.

The ZL10-250 A ultrasonic cleaner (Shanghai Zuo Le Instrument Co., Ltd.) was utilized to facilitate OP solubilization. UV-Vis spectra were measured using a UV-670 device (Shanghai Meipuda Instrument Co., Ltd.). Fluorescence emission data were acquired on an F-380 fluorescence spectrophotometer (Tianjin Gangdong Science and Technology Co., Ltd.). Circular dichroism (CD) spectra were recorded on a Chirascan Plus CD spectrometer (Applied Photophysics, Inc., U.K.). The solutions were thermostated using an SHZ-82 A water bath and a constant temperature oscillator (Changzhou Jintan Jingda Instrument Manufacturing Co., Ltd.).

### Experimental section

#### Fluorescence spectrometry

A 3 mL solution of BSA (1 µM) was added to a 1-cm cuvette, and the solution was thermostated at 298 K. To this solution, 30 µL of the OP stock solution (1 mM) was added sequentially for 9 times, and the emission spectra of the OP-BSA adduct were collected after each addition of OP. The *λ*_ex_ was configured at 280 nm, and fluorescence intensities were recorded within the range of 300–500 nm, employing a scan rate of 1200 nm/min. The slit widths were adjusted to 5 nm. The same steps were repeated at two additional temperatures, i.e., 293 K and 303 K.

### Synchronous fluorescence spectroscopy (SFS)

The SFS data of BSA and the OP-BSA complex at Δ*λ* = 15 and 60 nm (Δ*λ* = *λ*_em_-*λ*_ex_) were measured at 298 K between 250 nm and 350 nm. These experiments provided insights into the contribution Trp and Tyr residues in the binding of OP to BSA.

### 3D fluorescence spectroscopy

The 3D fluorescence spectra were recorded at a 1:1 (OP: BSA) mol ratio. Excitation wavelengths were scanned from 200 to 500 nm, and emission intensities were monitored from 200 to 600 nm, applying 10-nm increment.

### Fluorescent probe displacement

In a 1-cm cuvette containing 3 mL of BSA solution (1 µM) was added along with 3.0 µL of ibuprofen stock solution (1.0 × 10^− 3^ M). Fluorescence spectra were collected by sequentially adding 3.0 µL the OP solution (0.01 M) eight times in total. The temperature was adjusted to 303 K, and the fluorescence spectra of the OP-BSA system were scanned in the range from 300 to 500 nm. The same experiment was repeated using a 0.1 mM warfarin solution. All other test parameters remained the same as for fluorescence spectra acquisition.

### Ultraviolet (UV) spectrometry

To a 1-cm quartz cuvette, 3.0 mL of BSA solution (1 µM) was added, and 3.0 mL of PBS was used in the reference cell. The 3.0 µL of OP solution (0.1 mM) was added to this cuvette and the reference cell three times, and the UV absorption spectrum was scanned after each aliquot. The temperature was set to be 296 K, and the UV data were collected in the range of 250–350 nm.

### Circular dichroism (CD) spectra

A background scan of the PBS buffer solution was conducted using a circular dichroism (CD) spectrometer within a wavelength range from 200 to 260 nm. Subsequently, BSA solution in PBS (1 µM) was added to a 1-mm quartz cuvette, and the CD spectrum of BSA was recorded. Next, OP-BSA (concentration ratio equal to 2:1) was added, and the CD spectrum was scanned three times. The final CD spectra of pure BSA and OP-BSA adduct were obtained by subtracting the PBS background signal.

### Molecular docking

The BSA coordinates were retrieved from the Protein Data Bank (PDB ID 4F5S), while the initial conformation of OP was downloaded from PubChem. First, water molecules and irrelevant heteroatoms were removed using Pymol, retaining only the protein A chain. Amino acid pK_*a*_ values were calculated, and protonation states under neutral conditions were assigned using the propka3 online tool. The structures of the ligand and receptor were prepared for docking utilizing Autodock Tools-1.5.7 software. Molecular docking experiments were conducted using Watvina software (https://github.com/biocheming/watvina), specifying a square box with side lengths of 30 Å and a grid spacing step of 0.375 Å. The maximum limit for searching for conformations was set to 10,000, and conformational sampling and scoring were performed using a genetic algorithm. Optimal conformations were selected by sorting the conformations according to the docking scores.

### ADMET analysis

A computational ADME (absorption, distribution, metabolism and excretion) profile was generated for OP prior to its consideration for preclinical trials and in vivo studies. The analysis was conducted using the SwissADME web platform (http://www.swissadme.ch/) [31]. The toxicity assessment of OP was performed utilizing ADMETLAB 3.0 (https://admetlab3.scbdd.com/ ) [32].

## Results and discussion

### Fluorescence spectroscopy

#### Mechanism of fluorescence quenching by OP in BSA

Protein fluorescence quenching involves reducing the intrinsic fluorescence intensity, of the of proteins, primarily derived from the phenylalanine, tyrosine and tryptophan residues, which frequently participate in interactions with ligands [33]. Tryptophan contributes the most to the fluorescence, followed by tyrosine, while phenylalanine has minimal fluorescence and is considered insignificant compared to the other two [34]. Different physicochemical mechanisms may lead to fluorescence quenching, including energy transfer, collisions, formation of complexes in the ground state, or reactions in the excited state [35]. There are two primary types of quenching: static and dynamic. Their differentiation relies on their association with viscosity, temperature, and fluorescence lifetime. Static quenching occurs when a fluorescent substance and a quencher form a ground-state complex, while dynamic quenching occurs when the excited fluorophore relaxes to the ground state via collisions with the quencher [36].

**Fig. 2:**
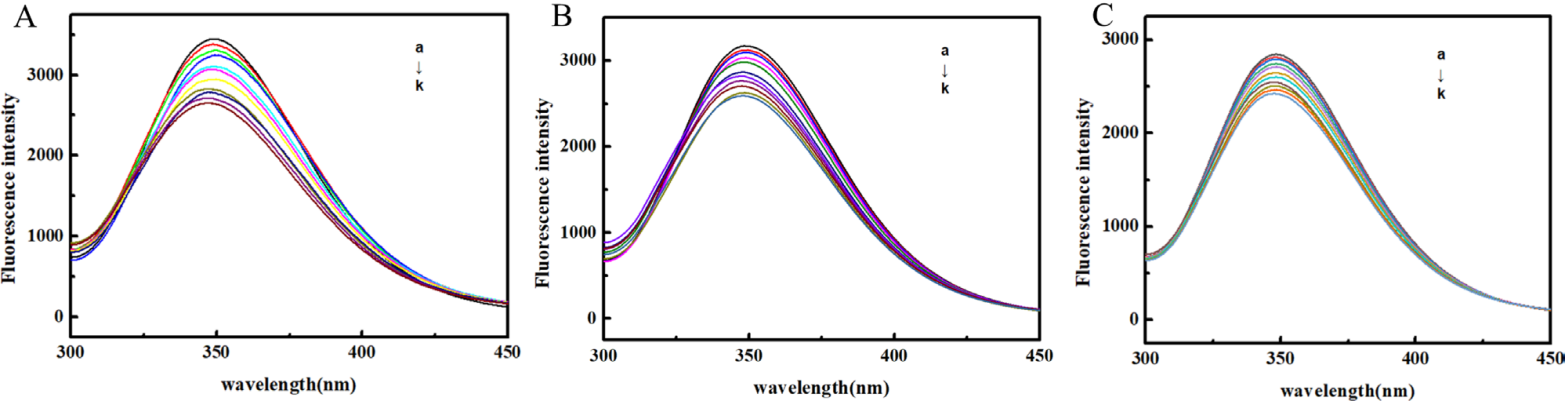
Fluorescence quenching spectra of BSA by OP at 293 K (**A**), 298 K (**B**) and 303 K (**C**) (C_BSA_ = 1.0 × 10^− 6^ mol∙L^− 1^ decreases from a to k, the OP concentration increases from 0 to 1.0 × 10^− 4^ mol∙L^− 1^, with a step size of 1.0 × 10^− 5^ mol∙L^− 1^)

The influence of OP on the emission intensity (*F*) of BSA is depicted in Fig. 2. With the addition of OP, the value of *F* decreased, and the maximum emission wavelength was blue-shifted, indicating that the microenvironment around BSA fluorophores became more hydrophobic upon OP binding. The changes in *F* values were further analyzed using the Stern-Volmer (*S-V*) model [37]:

1 $$\frac{{{F_0}}}{F} = 1 + {K_{SV}}\left[ Q \right] = 1 + {k_q}{\tau _0}\left[ Q \right]$$

where *F* and *F*_0_ represent emission intensity of the system in the presence or absence of OP, and *K*_SV_, *k*_q_, [*Q*] and *τ*_0_ represent the Stern–Volmer constant, bimolecular quenching rate constant, the molarity of OP, and the average fluorescence lifetime of biomolecules, respectively.

The *S-V* equation is a common tool for distinguishing between the static and dynamic quenching mechanisms. In the case of dynamic quenching, *K*_SV_ is positively correlated with temperature since the diffusion coefficients and the number of collisions increase with temperature. On the other hand, *K*_SV_ is negatively correlated with temperature in the case of static quenching, as temperature promotes the dissociation of ground-state complexes [38].

**Fig. 3:**
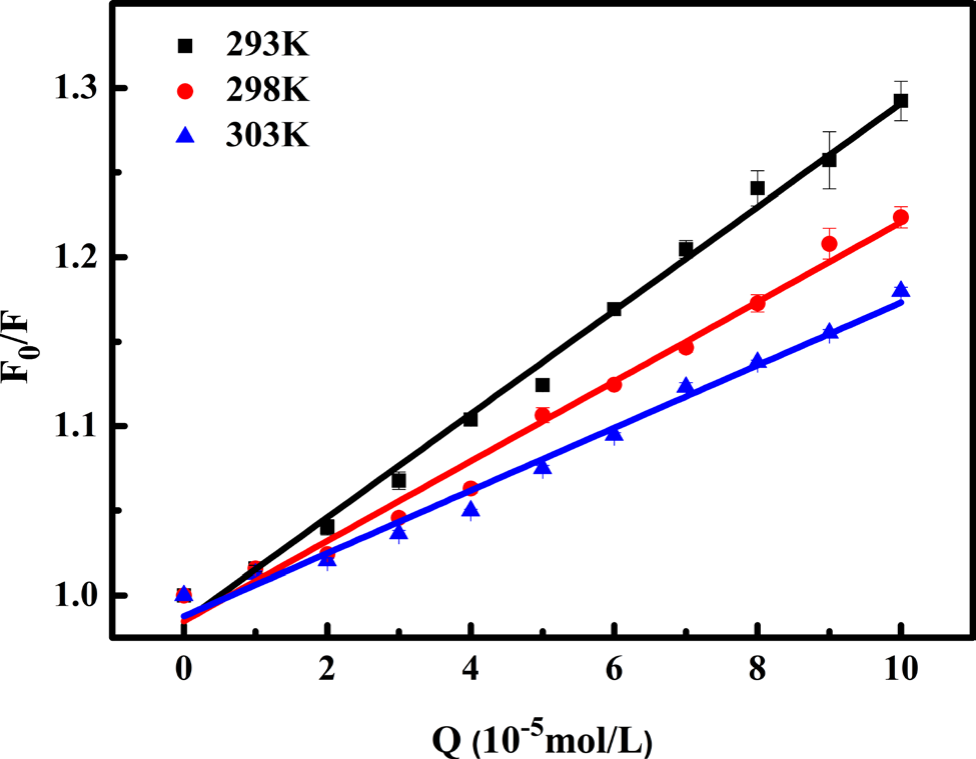
Stern-Volmer plots of the reactions of OP with BSA at different temperatures

Figure 3 shows the plot of *F*_0_/*F* against [*Q*] at three temperatures. The corresponding *K*_SV_ values, representing the slope of this trendline, are displayed in Table 1. The observed decrease in *K*_SV_ with rising temperatures suggests the dominance of the static quenching mechanism, attributed to the formation of the OP-BSA adduct. Furthermore, the *k*_q_ values, exceeding the diffusion limit for the dynamic quenching rate constant (2.0 × 10^10^ L·mol^− 1^·s^− 1^), provide additional confirmation for this result [39].

**Table 1:** Quenching constants of OP and BSA reactions at different temperatures

T/K	*R*	K_sv_(10^3^L·mol^− 1^)	k_q_(10^11^L·mol^− 1^·s^− 1^)	SD
293	0.99	3.06	3.06	0.08
298	0.99	2.36	2.36	0.09
303	0.98	1.86	1.86	0.07

#### Double logarithmic plot of the S-V model

Additional insights into the impact of OP on BSA were gained using the double logarithmic form of the *S-V* equation (Eq. 2) ^[40]^, providing information on the binding constant (*K*) and the number of binding sites (*n*).

2 $$\lg \left[ {\frac{{\left( {{F_0} - F} \right)}}{F}} \right] = \lg K + n\lg \left[ Q \right]$$

**Fig. 4:**
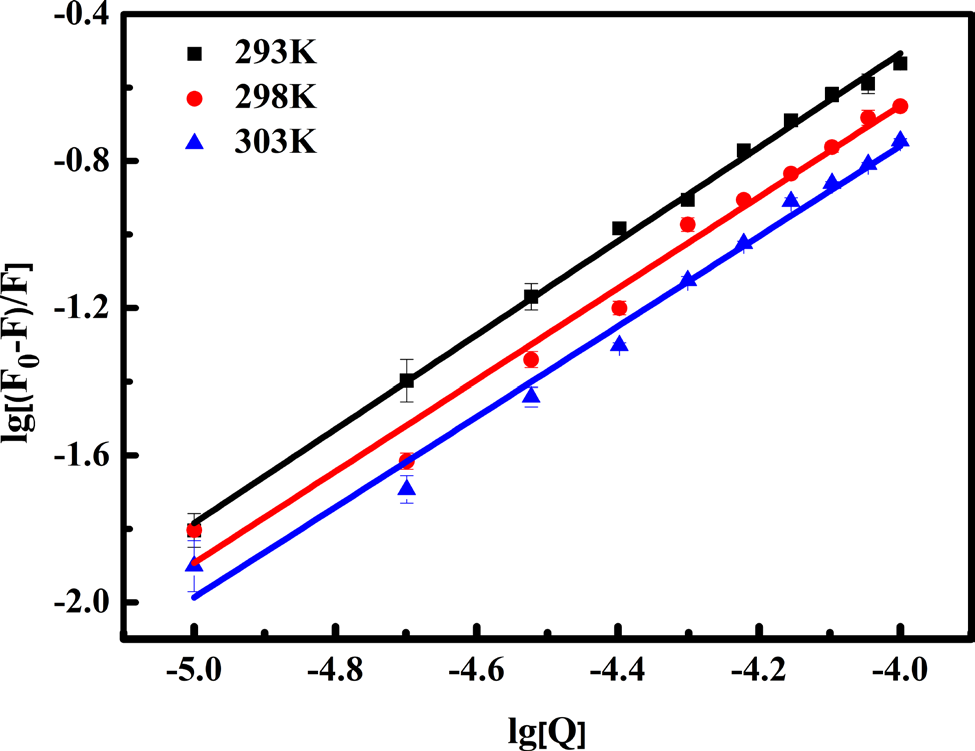
Double logarithmic curve of the reaction between OP and BSA

In this equation, [*Q*] represents the concentration of OP. The double logarithmic plots are presented in Fig. 4, and the corresponding values of slope (*n*) and intercept (log*K*) are listed in Table 2. The findings indicate that OP binds to a single binding site among many accessible sites on BSA. Moreover, as the temperature increases, *K* values decrease, suggesting the formation of the OP-BSA adduct rather than collision quenching.

**Table 2:** Binding constants and thermodynamic parameters for the reactions of OP with BSA at different temperatures

T(K)	K (10^4^ L∙mol^− 1^)	*R* ^a^	*n*	SD^b^	∆G (KJ∙mol^− 1^)	∆S (J∙mol^− 1^∙K^− 1^)	∆H (KJ∙mol^− 1^)
293	4.03	0.99	1.28	0.10	-25.76	-176.54	-77.49
298	2.09	0.98	1.24	0.26	-24.88		
303	1.41	0.98	1.22	0.23	-23.99		

^a^ Linear correlation coefffcient for the *K* values. ^b^ Standard deviation for the *K* values

#### Probe displacement experiments

**Fig. 5:**
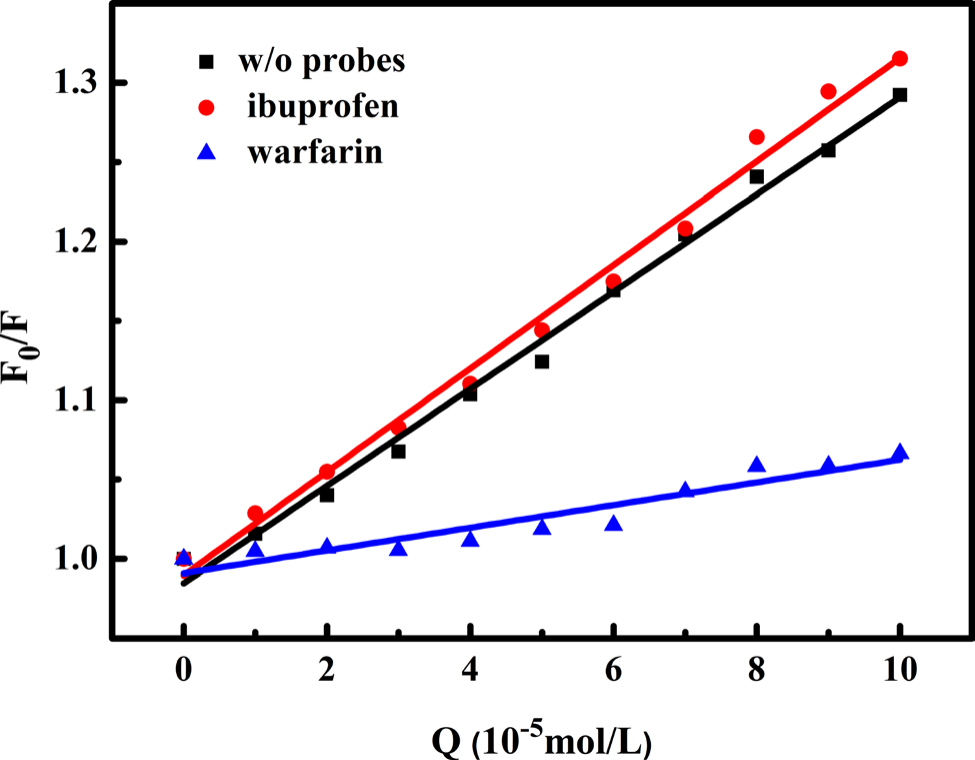
Stern-Volmer plot for the fluorescence of BSA-OP system in the absence and presence of site probes (warfarin and ibuprofen). C_BSA_ = C_probe_ =1 × 10^− 6^ mol∙L^− 1^

To determine the preferable binding sites for OP on BSA, we performed probe displacement binding assays using site-specific probes for binding site I (warfarin) and binding site II (ibuprofen) [40]. Figure 5 shows the Stern-Volmer plots of the BSA-OP system without and with site labeling at 303 K. The resulting values are listed in Table 3. We observed that the *K*sv values of the BSA-OP system all decreased in the presence of warfarin, suggesting that BSA-OP competes with warfarin for the same binding i.e. for Site I, and that the *K*sv values of the BSA-OP binding to BSA *K*sv values of site II did not change much in the presence of ibuprofen. This confirms that BSA-OP binds near site I, which is located in BSA subdomain IIA.

**Table 3:** Effect of site probes (warfarin and ibuprofen) on the interaction of OP with BSA

T(K)	K_sv_ w/o site probes	K_sv_ with ibuprofen	K_sv_ with warfain	*R* ^2^
303 K	3.06 × 10^3^	3.26 × 10^3^	7.15 × 10^2^	0.99/0.99/0.90

#### The thermodynamics of OP-BSA binding

The stabilization of the OP-BSA complex may result from various non-covalent interactions, such as hydrophobic forces, van der Waals forces, electrostatics, hydrogen bonding, and salt bridges [41]. According to Ross and Subramanian [42], the enthalpy change (Δ*H*) and entropy change (Δ*S*) determine the preferable type of interactions between compounds and biomolecules. These thermodynamic data can be calculated from the Van’t Hoff model (Eqs. 3 and 4):

3 $$\ln K = \frac{{ - \Delta H}}{{\left( {RT} \right)}} + \frac{{\Delta S}}{R}$$

4 $$\Delta G = \Delta H - T\Delta S$$

**Fig. 6:**
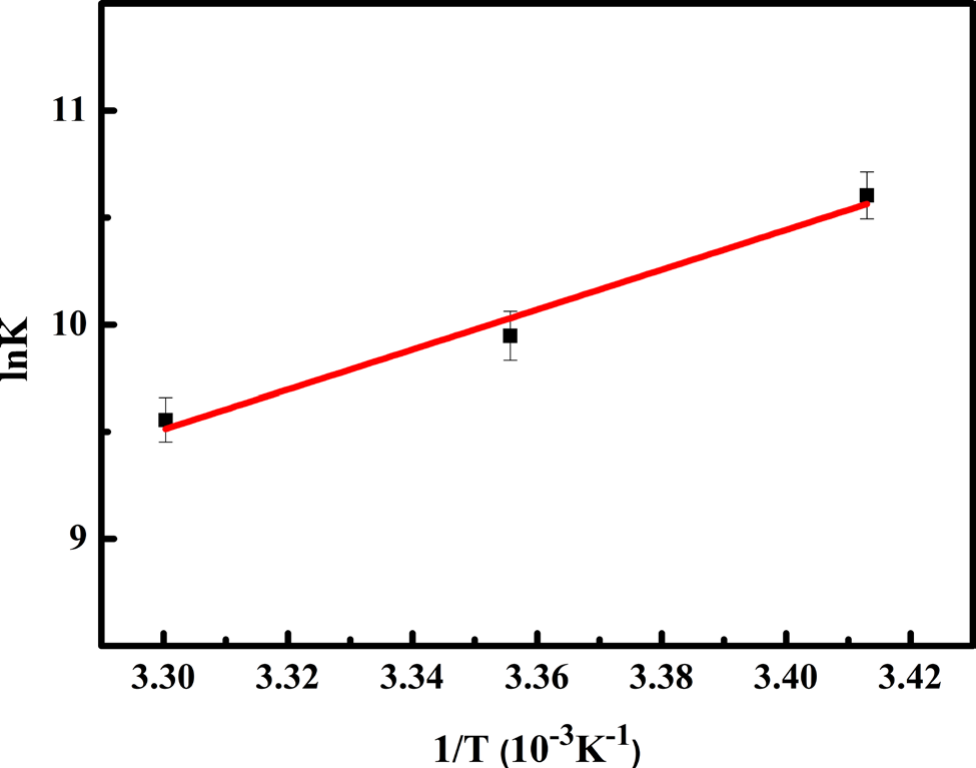
Van’t Hoff plots for the reaction of OP with BSA

In these equations, *K* represents the binding constant at temperature *T*, ∆*G* is Gibbs free energy change of the bonding process, and *R* is universal gas constant. From the linear plot of ln*K* vs. 1/*T* (Fig. 6), we obtained a ∆*H* value of -77.49 kJ∙mol^− 1^ from the slope and a ∆*S* value of -176.54 J∙mol^− 1^∙K^− 1^ from the intercept (see Table 2). Moreover, Gibbs free energy was negative for all temperatures, indicating the spontaneity of OP binding to BSA. These results imply that hydrogen bonding and van der Waals interactions are the dominant forces governing the binding process.

### Synchronous fluorescence spectroscopy (SFS)

**Fig. 7:**
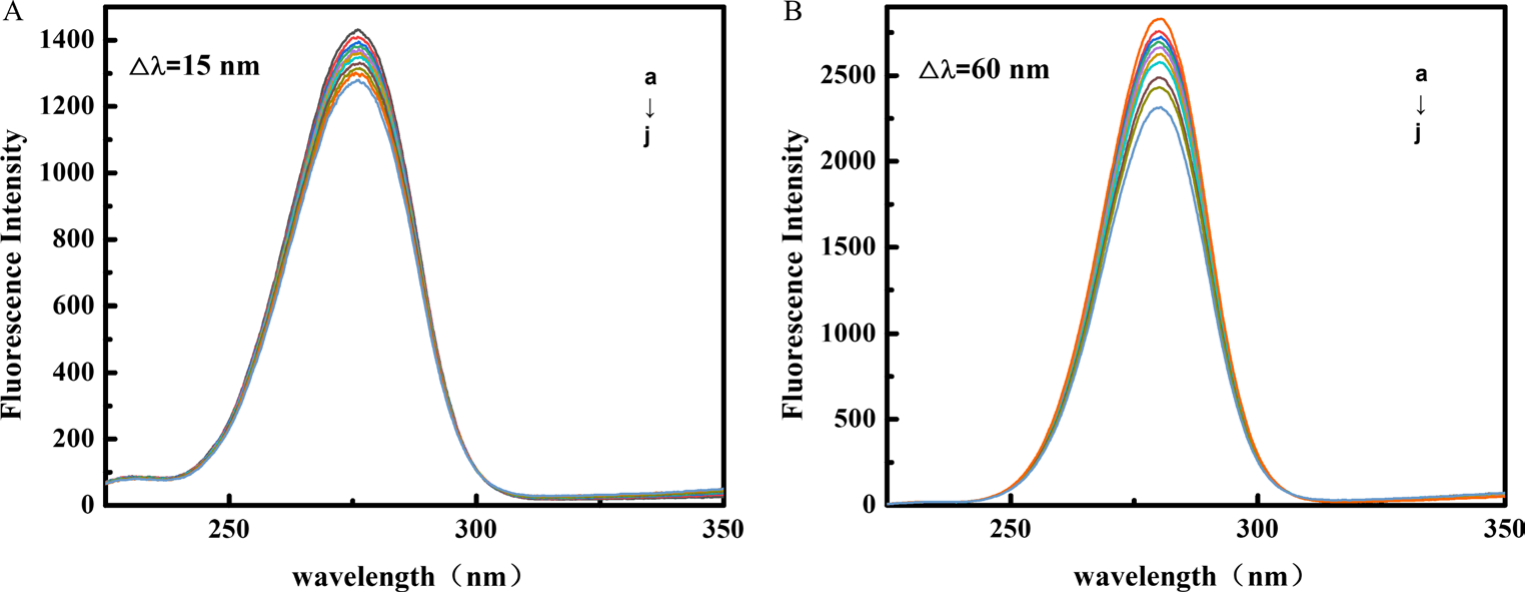
Synchronous fluorescence spectra of the interaction between OP and BSA at Δ*λ* = 15 nm (**A**) and Δ*λ* = 60 nm (**B**). (C_BSA_ = 1.0 × 10^− 6^ mol∙L^− 1^ decreases from a to j, the OP concentration increases from 0 to 9.0 × 10^− 5^ mol∙L^− 1^, with a step size of 1.0 × 10^− 5^ mol∙L^− 1^)

The SFS provides insights into the changes in the polarity near Trp and Tyr, the main amino acid residues contributing to the fluorescence emission of BSA [43]. According to Miller’s theory, setting Δ*λ* = 15 nm and Δ*λ* = 60 nm allows SFS to reveal changes in the microenvironment around Tyr and Trp caused by drug molecule binding [44]. The SFS data of BSA after OP addition at Δ*λ* = 15 nm and Δ*λ* = 60 nm are displayed in Fig. 7(A) and 7(B), respectively. The fluorescence intensity of BSA decreased gradually with increasing OP and the quenching at Δ*λ* = 60 nm was more pronounced than that at Δ*λ* = 15 nm, suggesting that OP binds more tightly to tryptophan residues than to tyrosine residues. In addition, there was little change in *λ*em for both (<1 nm), suggesting that the addition of OP had little effect on the microenvironment of Trp and Tyr residues.

### 3D fluorescence spectroscopy (3D-FS)

**Fig. 8:**
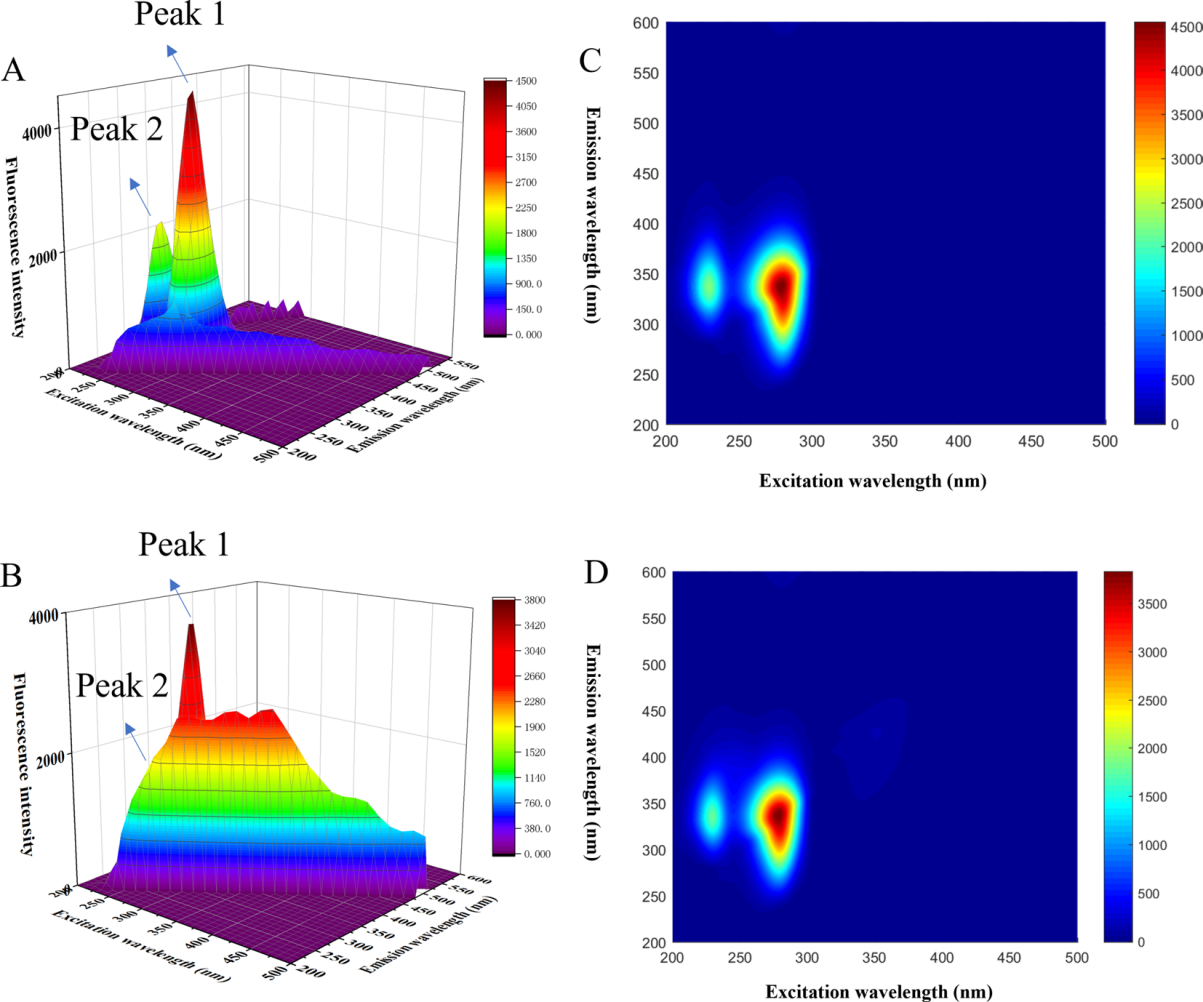
3D fluorescence and contour maps of AC BSA, BD OP-BSA. C_BSA_ = 1.0 × 10^− 6^ mol∙L^− 1^, C_OP_ = 1 × 10^− 5^ mol∙L^− 1^

The 3D-FS serves as a valuable tool for acquiring information about the microenvironment around Tyr and Trp, as well as the conformation of the peptide backbone [45]. The changes in 3D-FS data of BSA induced by OP binding are presented in Fig. 8. In Fig. 8 (A-B), the 3D emission spectra and contour plots of BSA and OP-BSA are depicted. Figure 8A shows two major peaks in the spectra of BSA and OP-BSA complex. The peak1 at λ_ex_ = 280 nm corresponds to the fluorescence emission of Tyr and Trp [46]. Conversely, peak 2 at λ_ex_ = 230 nm provides structural information about the peptide backbone of BSA [47]. Upon the addition of an equimolar amount of OP to BSA, the intensities of both peaks significantly decreased. The Stokes shift of peak 1 decrease from 4500 to 3791. upon the addition of OP, suggests that the microenvironment of Tyr and Trp in BSA became more hydrophobic upon ligand binding. Moreover, the decrease from 2218 to 1750, along with a 10 nm blue shift of peak(340 –330 nm), indicating conformational changes of the BSA peptide backbone and, consequently, alterations in the secondary structure of the entire BSA [48].

### UV absorption spectroscopy

**Fig. 9:**
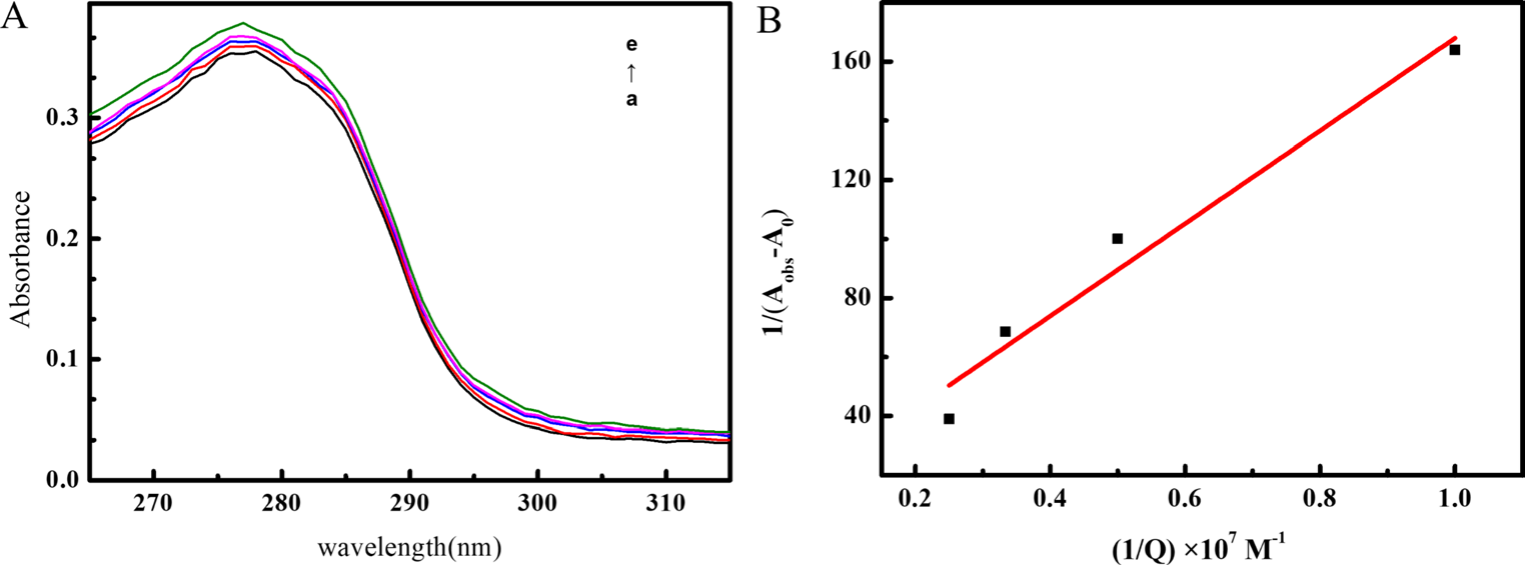
UV absorption spectra of OP and BSA (9 A) and Plot 1/(A_obs_-A_0_) versus1/[OP] (9B). (C_BSA_ = 1.0 × 10^− 6^ mol∙L^− 1^ decreases from a to e, the OP concentration increases from 0 to 4.0 × 10^− 7^ mol∙L^− 1^, with a step size of 1.0 × 10^− 7^ mol∙L^− 1^)

UV spectroscopy is a common technique to investigate protein-ligand interactions [49]. Figure 9 presents the UV absorption spectra of BSA after the addition of increasing amounts of OP. The absorption maximum at 277 nm is attributed to the π-π* electronic transitions in the aromatic residues of BSA, such as Tyr and Trp [50]. The results indicate that the peak shape and position remained unchanged upon the introduction of OP, but the peak intensity slightly increased. This observation further confirms the static quenching mechanism during OP-BSA complex formation, influencing both the microenvironment around fluorophores and the backbone conformation. Additionally, based on the absorbance spectra of BSA binding to OP, the intrinsic binding constant (*K*_b_) was calculated as follows (Eq. 5).

5 $$\frac{1}{{{A_{obs}} - {A_0}}} = \frac{1}{{{A_c} - {A_0}}} + \frac{1}{{{K_b}\left( {{A_c} - {A_0}} \right)\left[ {OP} \right]}}$$

*A*_obs_ was the absorbance of BSA with varying concentrations of OP. *A*_0_ was the absorbance of BSA alone and *A*_c_ the absorbance of OP-BSA complex. Based on the absorbance values, the relationship between 1/(*A*_obs_-*A*_0_) and 1/[OP] was plotted (Fig. 9B). A graph of 1/(*A*_obs_-*A*_0_) versus 1/[OP] yielded a linear plot with a slope equal to 1/*K*_b_(*A*_c_-*A*_0_) and an intercept equal to 1/(*A*_c_-*A*_0_). It was found in the plot that the value of *K*_b_ was 7.12 × 10^5^ M^− 1^.

### Circular dichroism (CD)

**Fig. 10:**
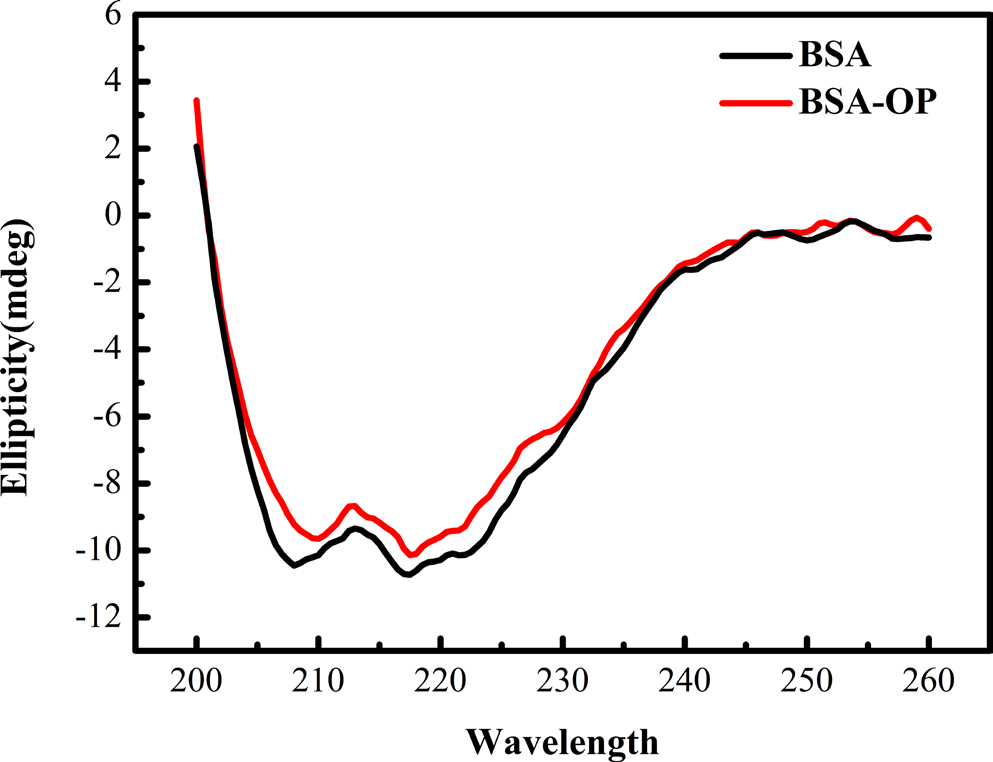
Circular dichroism spectra of BSA and BSA-OP (1:2) systems C_BSA_ = 1.0 × 10^− 6^ mol∙L^− 1^, C_OP_ = 2 × 10^− 6^ mol∙L^− 1^

CD is another common tool for studying how the binding of small molecules affects protein conformation [51]. In this study, we employed CD to investigate the influence of OP on the structure of BSA. The CD spectrum of BSA (Fig. 10) displays two negative peaks at 208 nm and 222 nm that originate from the π-π* and n-π* transitions in the α-helices. The intensity of these peaks reflects the degree of helicity in BSA. The percentage of α-helical secondary structure can be calculated using the following equation [52]:

6 $$MRE = \frac{{{\theta _{obs}}\left( {m\deg } \right)}}{{\left( {10 \times n \times l \times Cp} \right)}}$$

Here, MRE is the ellipticity of the average residue, θ_obs_ is the ellipticity, n is the number of amino acid residues, l represents the diameter of the cuvette, and Cp denotes the molarity of BSA. The α-helical content is derived from the ellipticity of the CD spectrum at 208 nm:

7 $$\alpha {\text{\% helix}} = \left[ {\frac{{\left( {{\text{ - }}MR{E_{208}} - 4000} \right)}}{{\left( {33000 - 4000} \right)}}} \right] \times 100$$

Calculated from Eqs. (6) and (7), the percentage of α-helical structure in BSA was 48.08%, and after the addition of OP (Fig. 10, red curve), this content decreased to 40.95%. This result confirms that OP binding induced changes in the secondary structure of BSA.

### Molecular docking simulations

**Fig. 11:**
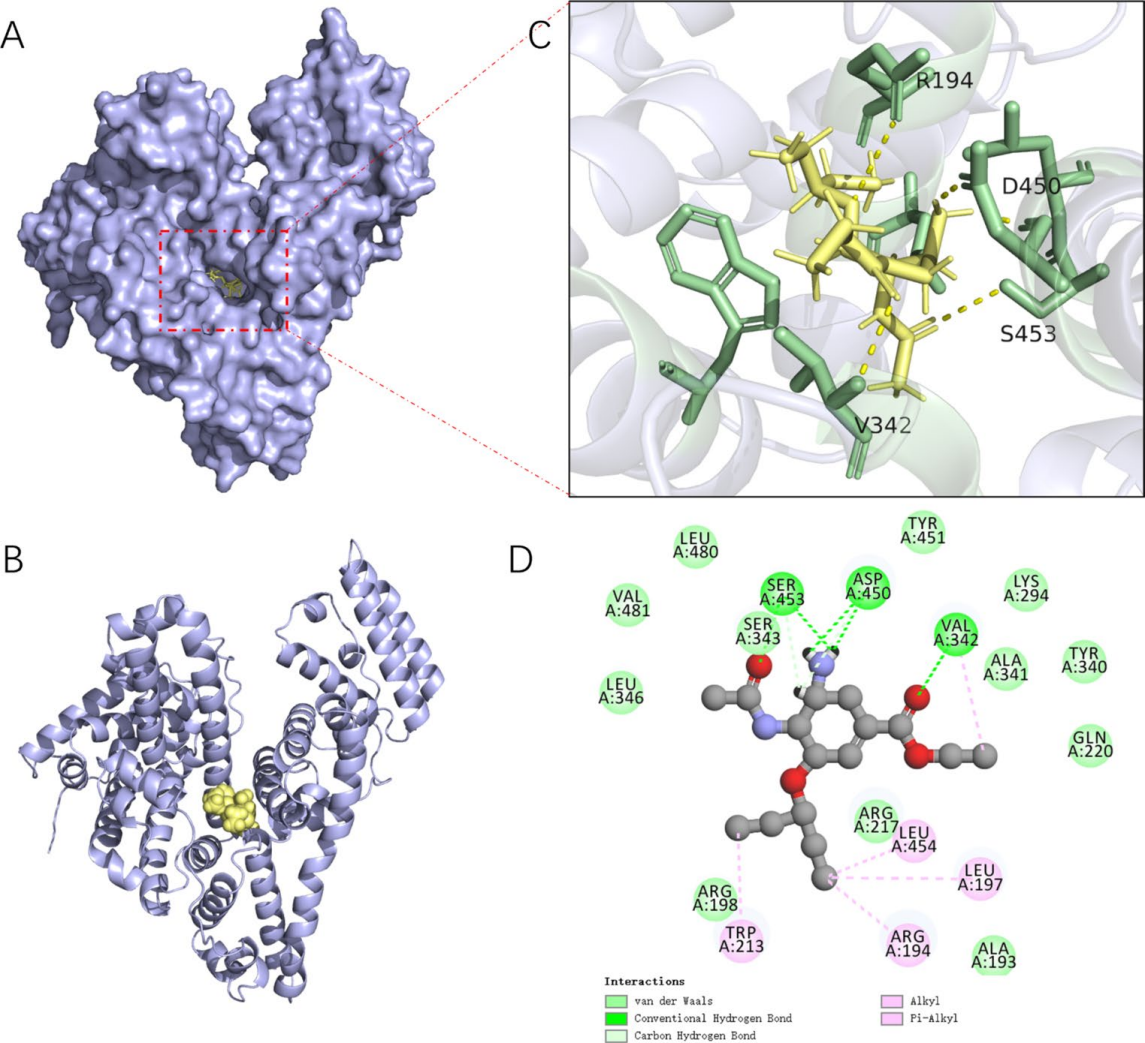
Analysis of BSA-site I and OP binding interface interactions (**A** and **B** are surface and ribbon structures, **C** and **D** are 3d and 2d plots of complex interface interactions)

To gain deeper insights into the binding mode of OP to BSA and identify crucial amino acids involved in ligand binding, molecular docking calculations were performed [54]. Figure 11 shows the binding mode of OP into site I of BSA. The ligand exhibits excellent shape complementarity with the binding site and a favorable binding affinity of − 5.96 kcal/mol. Furthermore, the ligand forms hydrophilic (electrostatic) interactions with the receptor binding site.

As illustrated in Fig. 11D, OP interacts with 19 amino acid residues of BSA. Among them, there are van der Waals forces between OP and LEU346, VAL481, LEU480, TYR451, LYS294, TYR340, GLN220, and VAL342, SER453, and ASP450, and seven hydrogen bonds are formed between OP and VAL342, SER453, ASP450. Five of them were conventional H-bonds formed between H atoms bound to highly electronegative atoms found at distances of 2.75 Å, 2.76 Å, 1.82 Å, 1.97 Å, 3.0 Å respectively. Additionally, two weaker interactions involving C-H bonds were found at distances of 2.98 Å and 2.08 Å. These results confirm the predominance of hydrogen bonding interactions in the stabilization of OP-BSA complex, consistent with the data derived from the temperature dependence of fluorescence spectra. Moreover, ARG194, LEU197, LEU454, VAL342 and TRP213 establish several hydrophobic interactions with OP. Since TRP213 is the main fluorophore in BSA, the interaction with this amino acid residue may explain the observed decrease in BSA emission intensity. Additionally, molecular docking suggests the formation of alkyl-alkyl and π-alkyl interactions between OP and TRP213, ARG194, LEU454 and LEU197 residues of BSA, further contributing to the stabilization of the OP-BSA complex.

### ADMET properties

The ADME properties of drugs were assessed using the SwissADME server. The investigation involved a comprehensive evaluation of OP, with a specific focus on its compliance with Lipinsk’s rule of five, encompassing parameters such as molecular weight below 500 g/mol, less than 10 hydrogen bond acceptors, and fewer than 5 hydrogen bond donors [53]. Findings from the supplementary materials in Table S1 revealed that OP did not violate any of the rules, suggesting potential orally active. Additionally, the consensus Log Po/w (the logarithm of the n-octanol/water distribution) value of OP was determined to be -0.17, indicating moderate lipophilicity. Moreover, SwissADME estimated the molar solubility of OP in water to be 2.47 by calculating the Log S (Silicos-IT) descriptor, indicating favorable water solubility [54]. Topological polar surface area (TPSA) of OP was found to be 178.22 Å, exceeding 140 Å, suggesting suboptimal permeability through various biological barriers [55]. OP showed no inhibitory effects on CYPs (1A2, 2C19, 2C9, 2D6, and 3A4) but exhibited inhibition of the human P-glycoprotein transporter (Pgp). Furthemore, the LogKp value of the compound was calculated to be -11.13 cm/s, indicating low skin permeability [56]. The Bio-availability Score of 55% suggested favorable bioavailability.

The potential toxicity of OP was assessed using ADMETLAB3, and the results were displayed in the Supplementary materils in Table S2. The findings revealed that OP did not inhibit the hERG (human ether-a-go-go-related gene) potassium channel, indicating it produced no cardiac toxicity or side effects [57]. The Rat Oral Acute Toxicity estimate for OP was 235 mg/kg, which can be classified to Class 2 (50 mg/kg < LD50 ≤ 500 mg/kg), thus it was non-toxic [60]. Additionally, the predicted Carcinogenicity Descriptor (CARC) value of 303 mg/kg body weight per day suggested that OP was unlikely to be carcinogenic. Consequently, OP was deemed non-carcinogenic and non-AMES toxic, enhancing its safety profile for potential pharmaceutical applications. To conclude, these results demonstrated that OP possessed favorable pharmacokinetic properties, making it suitable for oral administration and human consumption.

## Conclusion

In this study, we systematically assessed the interactions between OP and BSA using a combination of multiple spectroscopic methods and molecular docking simulations. The fluorescence spectroscopy results demonstrated that the addition of OP leads to the quenching of intrinsic fluorescence in BSA, primarily via a static quenching mechanism. Our investigation further revealed that OP exclusively binds to a specific binding site on BSA, and the displacement of the warfarin probe confirmed this site to be BSA site I. The thermodynamic analysis of the OP-BSA binding process indicated negative values for ∆*H*, ∆*S* and ∆*G*. These findings strongly suggested a spontaneous process driven by hydrogen bonding and van der Waals forces. Synchronous fluorescence spectra indicated that OP interacts with tryptophan residue of BSA. Furthermore, UV and CD spectroscopy indicated that the formation of OP-BSA complex induced changes in the protein secondary structure. Molecular docking calculations complemented our experimental results by validating that site I is the energetically most favorable binding site for OP on BSA. The docking also highlighted amino acid residues essential for OP-BSA interaction, and confirmed the dominance of hydrogen bonding and hydrophobic forces. Subsequently, an ADMET study forecasted favorable physicochemical and pharmacokinetic proffles for OP. This work contributes to a thorough understanding of the OP-BSA bonding mechanism, combining experimental and theoretical insights. The elucidation of these interactions is of considerable reference value for advancing research on the impact of OP on human health and furthering our understanding of the mechanisms underlying the action of antiviral drugs.

### Electronic supplementary material

Below is the link to the electronic supplementary material.

Supplementary Material 1

## Data Availability

The datasets used and/or analyzed during the current study are available from the corresponding author on reasonable request.

## Abbreviations

UV: Ultraviolet
OP: Oseltamivir phosphate
BSA: Bovine serum albumin
HSA: Human serum albumin
ADMET: Absorption, distribution, metabolism, excretion and toxicity
SFS: Synchronous fluorescence spectroscopy
3D-FS: 3D fluorescence spectroscopy
CD: Circular dichroism
Log Po/w: The logarithm of the n-octanol/water distribution
TPSA: Topological polar surface area
Pgp: P-glycoprotein transporter
CARC: Carcinogenicity descriptor
hERG: Human ether-a-go-go-related gene
