# Supplementary material for: Interaction mechanism of oseltamivir phosphate with bovine serum albumin: multispectroscopic and molecular docking study
Source: BMC Chem. 2024 Jul 5;18(1):126. doi: 10.1186/s13065-024-01232-0 (PMC11227190; doi:10.1186/s13065-024-01232-0)
Supplement: Supplementary file 1 — Supplementary Material 1 [file 13065_2024_1232_MOESM1_ESM.doc]

**Interaction Mechanism of Oseltamivir Phosphate with Bovine Serum Albumin:** **Multispectroscopic and** **Molecular Docking Study**

JingYu^1^, Jian-Ming Liu^1^, Hui-Yi Chen^1^, Wei-Ming Xiong^2^*

^1^School of Chemistry and Civil Engineering, Shaoguan University, Shaoguan, 512023, China

^2^School of Physical Science & Technology, Guangxi University, Nanning 530004, China

*Correspondence: Wei-Ming Xiong, xiongwm3@gxu.edu.cn

**SUPPLEMENTARY MATERIALS: Table of Contents**

**Table S1.**  ADME properties of OP

| Physcicochemical  properties | | Lipophilicity | | Water Solubility | | Pharmacokinetics | | Drug-likeness | |
| --- | --- | --- | --- | --- | --- | --- | --- | --- | --- |
| formula | C16H31N208P | Log Po/w  (ILOGP) | 1.71 | LogS (ESOL) | 0.27 | Pharmacokinetics GIA | LOW | Lipinsk | Yes |
| M.W | 410.40 g/mol | Log Po/w  (XLOGP3) | -3.27 | Solubility | 7.64e+02mg/ml;1.86e+00 mol/l | BBB permean | No | Ghose | Yes |
| Fraction Csp3 | 0.75 | Log Po/w  (WLOGP3) | 0.36 | Class | Highly soluble | P-gp substrate | Yes | Veber | No |
| NROT | 9 | Log Po/w  (MLOGP3) | -0.97 | LogS (Ali) | 0.10 | CYP1A2  inhibitor | No | Egan | No |
| HBA | 9 | Log Po/w  (SILICOS-IT) | 1.33 | Solubility | 5.16e+02 mg/ml ;1.26e+00 mol/l | CYP2C19  inhibitor | No | Muegge | No |
| HBD | 5 | Consensus Log Po/w | -0.17 | Class | Highly soluble | CYP2C9  inhibitor | No | Bio-availability Score | 0.55 |
| MR | 98.78 |  |  | LogS (SILICOS-IT) | 2.47 | CYP2D6  inhibitor | No |  |  |
| TPSA | 178.22A˚ |  |  | Solubility | 1.38e+00 mgiml ;3.37e-03 mol/l | CYP3A4  inhibitor | No |  |  |
|  |  |  |  | Class | Soluble | LogKp(skin permeation) | -11.13 cm/s |  |  |

M.W: molecular weight; NROT: No. of rotating bonds; HBA: No. of H-bond acceptors;. HBD: No. of H-bond donors; MR: Molar Refractivity; TPSA: Topological polar surface area. GIA: gastrointestinal absorption; BBB: blood-brain barrier;. CYP: Cytochrome P450; P-gp substrate: Glycoprotein substrate P.

**Table S2.**  Toxicity properties estimated for OP

| TOXICITY | | | ENVIREMENTAL TOXICTY | |
| --- | --- | --- | --- | --- |
| Property | Value | Decision | Property | Value |
| hERG Blockers | 0.232 | excellent | BCF | 0.483 |
| DILI | 0.814 | poor | IGC50 | 3.118 |
| AMES Muta genicity | 0.234 | excellent | LC50 | 3.866 |
| Rat Oral Acute Toxicity | 0.235 | excellent | LC50DM | 4.389 |
| FDAMDD | 0.671 | medium |  |  |
| Skin Sensitization | 0.893 | poor |  |  |
| Carcinogenicity | 0.303 | medium |  |  |
| Eye Corrosion | 0.001 | excellent |  |  |
| Eye Irritation | 0.099 | excellent |  |  |
| Respiratory | 0.723 | poor |  |  |
| Human Hepatotoxicity | 0.598 | medium |  |  |
| Drug-induced Nephrotoxicity | 0.36 | medium |  |  |
| Ototoxicity | 0.682 | medium |  |  |
| Hematotoxicity | 0.118 | medium |  |  |
